# SERS-based Immunoassay in a Microfluidic System for the Multiplexed Recognition of Interleukins from Blood Plasma: Towards Picogram Detection

**DOI:** 10.1038/s41598-017-11152-w

**Published:** 2017-09-06

**Authors:** Agnieszka Kamińska, Katarzyna Winkler, Aneta Kowalska, Evelin Witkowska, Tomasz Szymborski, Anna Janeczek, Jacek Waluk

**Affiliations:** 1 Institute of Physical Chemistry, Polish Academy of Sciences, Kasprzaka 44/52, 01-224 Warsaw, Poland; 2 Faculty of Mathematics and Natural Sciences, College of Science, Cardinal Stefan Wyszyński University, Dewajtis 5, 01-815 Warsaw, Poland

## Abstract

SERS-active nanostructures incorporated into a microfluidic device have been developed for rapid and multiplex monitoring of selected Type 1 cytokine (interleukins: IL-6, IL-8, IL-18) levels in blood plasma. Multiple analyses have been performed by using nanoparticles, each coated with different Raman reporter molecules: 5,5′-dithio-bis(2-nitro-benzoic acid) (DTNB), fuchsin (FC), and p-mercatpobenzoic acid (p-MBA) and with specific antibodies. The multivariate statistical method, principal component analysis (PCA), was applied for segregation of three different antigen-antibody complexes encoded by three Raman reporters (FC, p-MBA, and DTNB) during simultaneous multiplexed detection approach. To the best of our knowledge, we have also presented, for the first time, a possibility for multiplexed quantification of three interleukins: IL-6, IL-8, and IL-18 in blood plasma samples using SERS technique. Our method improves the detection limit in comparison to standard ELISA methods. The low detection limits were estimated to be 2.3 pg·ml^−1^, 6.5 pg·ml^−1^, and 4.2 pg·ml^−1^ in a parallel approach, and 3.8 pg·ml^−1^, 7.5 pg·ml^−1^, and 5.2 pg·ml^−1^ in a simultaneous multiplexed method for IL-6, IL-8, and IL-18, respectively. This demonstrated the sensitivity and reproducibility desirable for analytical examinations.

## Introduction

The immune system is composed of a variety of different cell types and proteins. Each element performs a specific task aimed at recognizing and/or reacting against ‘foreign material’. Disorders of the immune system can result in autoimmune diseases, inflammatory diseases, and cancer. There are more than 250 primary immunodeficiency diseases recognized by the World Health Organization. Therefore, development of new methods of quantitative analysis of immune markers will lead to a better understanding of immune system disorders and should open new avenues for therapeutic regimens.

Up to now several analytical procedures have been applied for evaluating cytokine levels in body fluids. Enzyme-linked immunosorbent assays (ELISA) have long been considered as the ‘gold standard’, but nowadays, there is a need to develop the multiplex technology which allows investigation of whole cytokine networks. Wang *et al*.^1, 2^ demonstrated detection of IL-6 and IL-8 interleukin from a buffer solution. Measurement of the levels of cytokines of immune activation can provide reliable information regarding a disease’s diagnosis, stage, prognosis, and the evaluation of therapy. However, difficulties and inaccuracies have been reported, and a number of factors have been shown to affect the validity and the quality of such measurements. Differences in the levels of measured analytes for identical samples in the range of 10- to 100-fold have been reported^3–5^. Also, because of the numerous biological activities of individual cytokines and the complex cytokine network, cytokines have not been employed in clinical laboratory tests.

Interleukins in body fluids usually circulate in tiny amounts (nano- and picomolar) and their concentrations can increase up to 1000-fold when immune system activation is required. Interleukin-6 (IL-6), a pleiotropic cytokine, is produced by various types of normal and cancer cells and is implicated in the stimulation of tumor cell proliferation, malignant transformation, and tumor progression^6^. Additionally, it has been reported that a high circulating IL-6 level might predict an inferior response to chemotherapy and therefore is an independent prognostic marker for breast cancer^7^ and lung cancer-specific survival, especially for those who receive chemotherapy^8^. Interleukin-8 (IL-8), an inflammatory cytokine, may also play an important role in breast cancer. So far, there have been only a few research papers concerning this cytokine. Yokoe *et al*. have measured serum IL-8 levels in 12 heavily pretreated patients with recurrent breast cancer and reported a small increase of IL-8 in those patients with refractory progressive disease and almost no decrease in those with partial response or no change after systemic therapy^9^. Interleukin-18 (IL-18) plays an important role in the T-cell-helper response. It acts as an angiogenic factor and a tumor suppressor. Some clinical studies have shown that the serum IL-18 level may be a prognostic factor in patients with gastric carcinoma, hematological malignancies, and metastatic breast cancer.

The particular interleukins IL-6, IL-8, and IL-18 are known to play a diverse role in breast cancer initiation and progression. However, only a few sensors for these immune markers have been proposed in the literature and until now there are no methods which allow the simultaneous quantification and multiplex analysis of interleukins in the body fluids. The development of such methods is extremely important. The complications caused by the presence of interfering compounds in a given sample form a major drawback in existing molecular sensor technologies, particularly in multi-analyte systems. SERS as a molecular fingerprinting technique has the ability to resolve analytes within mixtures. Current successes in the development of nanotechnologies and instrumentations have led to recognition of biomolecular systems based on SERS with higher sensitivity and chemical specificity^10–12^.

The SERS technology can be used potentially for the quantitative measurement of analytes with ultrahigh sensitivity and offers nondestructive, reliable, and fast detection of samples, which leads to various practical applications in studying, e.g. nucleic acids and proteins^13^, therapeutic agents^14^, drugs and trace materials^15^, and bacteria cells^16, 17^.

To the best of our knowledge, this is the first report on the efficient, multiplex, and sensitive surface-enhanced Raman scattering (SERS)–immunoassay in a microfluidic device for the detection of three selected interleukins: IL-6, IL-8, IL-18 in blood plasma. The strength of reported SERS-based detection lies in merging:

(i) a SERS-active platform based on a bimetallic Ag-Au surface that offers high sensitivity, reproducibility, and stability of the recorded SERS signals,
(ii) appropriately designed Raman reporters: basic fuchsin (FC), 5,5′-dithio-bis(2-nitro-benzoic acid) (DTNB), and *p*-mercaptobenzoic acid (*p*-MBA) that give strong SERS enhancement and have the ability to bind both antibodies and gold structures,
(iii) the constructed microfluidic device for multiplexed analysis in point, that additionally enhances the reproducibility of the SERS-based immunoassay method, and
(iv) principal component analysis (PCA) applied to develop diagnostic algorithms for improving the detection and discrimination of a particular interleukin in complex biological samples.

## Methods

### General procedure

**Figure 1:**
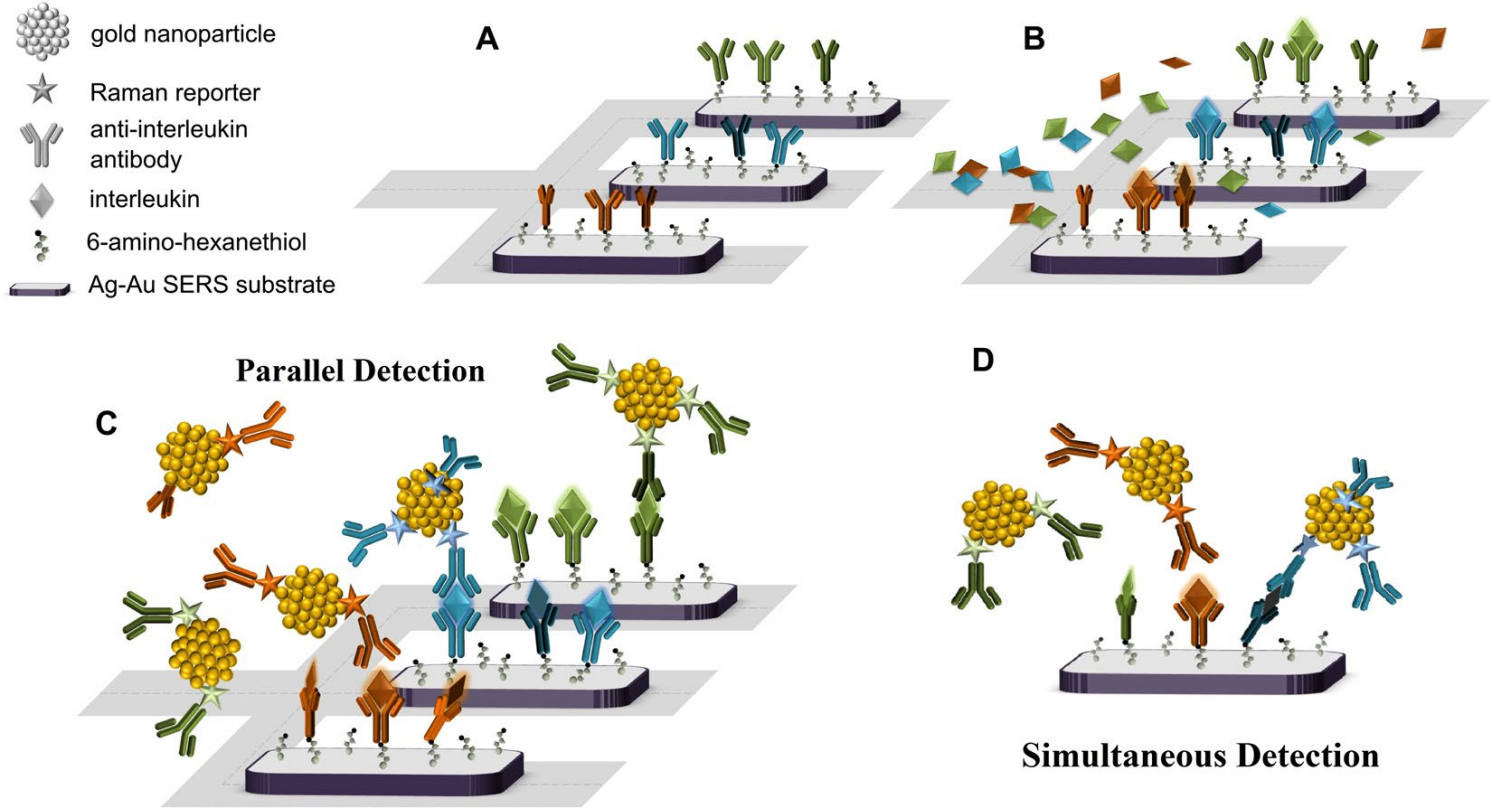
Sequential steps for the formation of a SERS-based multiplex immunoassay. (**A**) The capturing substrate, (**B**) the mixture of interleukins (IL-6, IL-8, IL-18) in human blood plasma injection, (**C**) the parallel approach (**D**) the simultaneous multiplex configuration.

The multiplexed analysis (named parallel and simultaneous) based on a sandwich type SERS immunoassay is schematically illustrated in Fig. 1. The first layer of this sandwich structure is composed of immobilized antibodies against IL-6, IL-8, and IL-18 interleukins captured on a Ag-Au bimetallic SERS-active surface via 6-amino-1-hexanethiol (AHT) layers. This particular SERS substrate fulfills all the necessary requirements for biological, medical and analytical analysis, such as high sensitivity, stability, biocompatibility, and reproducibility^18^. The second layer comprises the complementary interleukins (proteins) captured by the selective antibodies. The third layer consists of Raman reporters (FC, *p*-MBA, and DTNB)-and antibodies-labeled immune-Au-nanoparticles. In the parallel approach (Fig. 1C), the individual SERS-active platform was functionalized using antibodies against selected interleukins: anti-IL6, anti-IL8, and anti-IL18, respectively. A microfluidic SERS-device with three chambers allows parallel detection and analysis of three selected interleukin levels in blood plasma. In the simultaneous multiplexed detection method (Fig. 1D), the SERS-active platform was functionalized with three antibodies, anti-IL6, anti-IL8, and anti-IL18 to permit the simultaneous detection of three target interleukins within the platform. A detailed description of the performance of the microfluidic SERS device is presented in Supplementary Materials, Chapter 5.

### Ethical Statements

All experiments were performed in compliance with the relevant laws and institutional guidelines. The protocol of study was approved by the Ethics and Bioethics Committee of Cardinal Stefan Wyszyński University in Warsaw. Informed consent was obtained from all patients.

## Experimental

### Reagents

Recombinant human interleukin-8 (CXCL8) and monoclonal anti-Interleukin-8 antibody produced in mouse (clone 6217); interleukin-6 (recombinant, expressed in *E. coli*), monoclonal anti-IL-6 antibody produced in mouse (clone 3H6), anti-interleukin 18 antibody, (clone CPTC-IL18-1), and human interleukin-18 were purchased from Sigma and used as received. Basic fuchsin (FC), *p*-mercaptobenzoic acid (*p*-MBA), 5.5′-dithio-bis(2-nitro-benzoic acid (DTNB), L-ascorbic acid, gold (III) chloride trihydrate, trisodium citrate dihydrate, hexadecyltrimethylammonium bromide (CTAB), *N*-hydroxysulfosuccinimide sodium salt (NHS), 5.5′-Dithio-bis(2-nitrobenzoic acid) (DTNB), 1-ethyl-3-(3-dimethylaminopropyl) carbodiimide hydrochloride (EDC), 6-amino-1-hexanethiol (AHT), bovine blood plasma albumin (BSA), and phosphate-buffered saline (PBS) packs (10 mM, pH = 7.2) were obtained from Sigma.

### Fabrication of a microfluidic chip

The microfluidic chip has been designed using MasterCAM software and then micromachined with a computer numerical-controlled (CNC) milling machine (ErgWind, type MFG4025P) in a 5 mm polycarbonate (PC) slab (Bayer). The machined channels had 200 µm or 400 µm width and 350 µm depth. To join milled and plain PC slabs we pressed them together at high temperature (T = 130 °C) for 30 minutes. A high precision syringe pump system (Harvard Apparatus Pump Series, MA, USA) was used for automated control of flow. Standard polyethylene (PE) tubings with an inner diameter of 0.8 mm were used for interconnection of the chip at the syringe pump. The SERS-active nanostructures based onto a Ag–Au bimetallic SERS substrate (5 mm of diameter and 4 mm of thickness) were placed onto the detection area chambers (DA1-3) of the microfluidic chip (Fig. 1) to record SERS signals from this point. The detection points are open to air and the recorded SERS signals are not affected by the material of the microfluidic device.

### Capturing Substrate Preparation

#### Ag-Au bimetallic substrate synthesis

The Ag-Au bimetallic substrate was synthesized according to previously published procedures^17^. The silver disc electrodes were mechanically mirror-polished with alumina slurries with sequentially decreasing particle sizes (0.5 mm, 0.05 mm, and 0.02 mm). After each step of polishing, the electrodes were immersed in Millipore water and subsequently sonicated in an aqueous ultrasonic bath for 15 minutes in order to remove the physically adsorbed alumina particles from the electrode surface. After polishing, the electrodes were electrochemically roughened with an oxidation/reduction cycle (ORC). The electrochemical cell was filled with 0.1 M KCl solution and subsequently purged with argon gas for 30 min to remove most of the air from the solution. The rough silver substrate was prepared by three ORCs (0.5 V for 40 s and −0.5 V for 40 s; 0.5 V for 15 s and −0.5 V for 15 s, and to finish, 0.5 V for 15 s and −0.5 V for 30 s) and a final reduction potential of −0.4 V for 300 s. The electrode was then thoroughly washed with Millipore water and dried with a flow of argon. In the next step, the gold nanostructures were deposited over the electrochemically obtained silver surface. The three-electrode electrochemical cell was filled with a solution of 0.4 mM HAuCl_4_ in 0.1 M HClO_4_ and subsequently purged with argon for 30 min to remove most of the air from the solution. Then the potential of −80 mV was applied for 200 s. Subsequently, the silver-gold bimetallic substrates were washed in deionized water, dried in a stream of nitrogen, and placed in the microfluidic chambers (Fig. S6).

#### Ag-Au bimetallic substrate modification

The Ag-Au bimetallic SERS-active substrates were modified with a 0.1 mM ethanol solution of 6-amino-1-hexanethiol for 5 hours to form an amino-terminated linkage monolayer. Then, the amino-modified surfaces were completed by injecting mixtures of 5 µL of 100 µg·mL^−1^, 5 µL of 120 µg·mL^−1^, 5 µL of 80 µg·mL^−1^ anti-interleukin 6 (anti-IL-6), anti-interleukin-8 (anti-IL8), and anti-interleukin 18 (anti-IL18), respectively, and an activation solution (0.2 M EDC/0.05 M NHS; mixture in deionized water) in the molar ratio of 5:1 into the detection areas (chambers DA1-3 in Fig. S6). After 1 hour, the remaining active surface was blocked by injecting 5 µL of 2% BSA in PBS buffer solution (pH = 7.2) via inlet X (Fig. S6). In the next step, the samples ware rinsed twice with 5 mL of 10 mM PBS buffer solution via the same inlet O. Finally, the antibody-immobilized substrates were stored at 4 °C for future use.

### Preparation of the Raman reporter-labeled immuno-Au nanoparticles (anti-IL6 /AuNPs-FC; anti-IL8/AuNPs-P-MBA; anti-IL16 /AuNPs-DTNB)

For the multiplex SERS immunoassay, a set of Raman reporters (FC, *p*-PMBA, and DTNB-labeled immune-Au nanoparticles) were synthesized. For detailed procedures see Supplementary Materials. The average diameter of gold nanoparticles was about 70 nm according to the SEM images and the histogram of diameters of Au nanoparticles (Figs S2 and S3). Additionally, Fig. S4 shows the sequential steps for the formation of the Raman reporter-labeled immuno-Au nanoparticles.

### Blood samples preparation

In our experiments we used human blood samples from 10 healthy volunteers available by courtesy of the Regional Blood Center. The samples underwent morphological analyses prior to use and revealed no abnormalities. Informed consent was obtained from all patients. All experiments were performed in compliance with the relevant laws and institutional guidelines. The protocol of study was approved by the Ethics and Bioethics Committee of Cardinal Stefan Wyszynski University in Warsaw.

### Chemometrics

The SERS spectra were prepared for principal component analysis (PCA) using a two-step approach. First, using an OPUS software (Bruker Optic GmbH 2012 version) the spectra were smoothed with a Savitsky-Golay filter, the background was removed using baseline correction, and then the spectra were normalized using a so-called Min-Max normalization. Then all the data were transferred to Unscrambler software (CAMO software AS, version 10.3, Norway) where the PCA analysis was performed. PCA is a multivariate technique that reduces the dimensionality of complex spectroscopic data from many wavenumber assignments to a few principal components (PCs) making it easier to identify the majority of variations within the spectra^19^.

In the present work PCA was carried out on three different data sets, consisting of spectra obtained for three types of studied immunocomplexes encoded by three types of Raman reporters (FC, *p*-MBA, and DTNB).

### Immunoassay protocol

In the parallel approach (Fig. 1C and Fig. S6B), an individual SERS-active platform was functionalized using particular antibodies against selected interleukins - anti-IL6, anti-IL8, and anti-IL18, respectively. In this configuration, the inlets X2, Y2, and Z2 were used to functionalize each individual SERS-active platform with particular antibodies (via 6-amino-1-hexanethiols layers) to create the first layer of a sandwich structure of the SERS-based immunoassay. In the next step, PBS buffer solutions (pH = 7.2) were added via inlets X2, Y2, and Z2 to remove physically unadsorbed antibodies. Then, interleukins IL-6, IL-8, and IL-18 antibodies in human blood plasma (target proteins and infection markers) and Raman reporter-labeled immuno-Au-nanoparticles (anti-IL6/AuNPs-FC; anti-IL8/AuNPs-*p*-MBA; anti-IL16 /AuNPs-DTNB) were sequentially injected into inlets X_2_, Y_2_, Z_2_, and O_2,_ mixed, and then transferred via tubings H1-3 to the capturing Ag-Au SERS-active surfaces for the formation of sandwich immunocomplexes. The SERS-active substrates are incorporated in the detection areas (DA1-3, Fig. S6). These are the places where the sandwich immune-complexes are created and detected via the SERS technique.

In the simultaneous multiplexed detection method (Fig. 1D and Fig. S6A) the SERS-active platform was functionalized with three antibodies including anti-IL6, anti-IL8, and anti-IL18 to enable the simultaneous detection of three target interleukins on the same platform. The three different antibodies against IL-6, IL-8, and IL-18 interleukins were injected via inlets X1–3 in order to be captured (using EDC/NHS chemistry) on the 6-amino-1-hexanethiol (AHT) modified Ag-Au bimetallic SERS-active surfaces (DA1-3, Fig. S6). The same inlets were used to introduce the human serum samples with designed concentrations of interleukins. Subsequently, the captured interleukins were detected with respective Raman reporter-labeled Au nanoparticles injected via inlet O1.

Usually, in both parallel and simultaneous configurations the interleukins in human blood plasma samples at the proper concentrations were injected at the rate of 1.5 µL·min^−1^, whilst the mixture of three kinds of used Raman reporters FC, *p*-MBA, and DTNB was injected at the rate of 2.5 µL·min^−1^. To generate the appropriate sandwich immunocomplexes the flows were stopped for 10 min and all the reagents were incubated in the detection areas of the microfluidic device (chambers DA1–3 in Fig. S6). At the end, PBS buffer solutions (pH = 7.2) were again injected via inlets O (Fig. S6) to remove physically unadsorbed antigens and other reagents.

### Raman and SERS measurements

SERS measurements were performed using the Renishaw inVia Raman system equipped with a 632 nm He-Ne laser excitations source The light from the laser was passed through a line filter and focused on a sample mounted on an X-Y-Z translation stage with a × 10 microscope objective. The Raman-scattered light was collected by the same objective through a holographic notch filter to block out Rayleigh scattering.

An 1800 groove·mm^−1^ grating was used to provide a spectral resolution of 5 cm^−1^. The Raman scattering signal was recorded by a 1024 × 256 pixel RenCam CCD detector. The beam diameter was approximately 5 µm. Typically, the SERS spectra were recorded at 30 s integration time with 2.5 mW laser output power by mapping an area of 50 µm × 50 µm.

## Results and Discussion

### Characterizing the capturing of substrate and Raman reporter-labeled immune-Au nanoparticles

An important aspect of the present study is to create a SERS-active surface that supports a SERS enhancement factor (EF) substantial enough to detect the analyzed molecules–especially interleukins – at physiologically relevant concentrations. As shown in the previous work^17^, the Ag-Au bimetallic substrates display a peak SERS EF of 10^7^, and thus demonstrate the ability to perform detection of low-concentration standard analytes like *p*-mercaptobenzoic acid and biological samples like ABO antigens and antibodies^20^ or bacteria cells^21^. By applying the Ag-Au bimetallic substrate we have combined the characteristic features of both metals: the high chemical stability of Au along with very high Raman scattering enhancement for Ag. The SERS-active substrate used in this study exhibits, besides a uniformly high enhancement factor, high reproducibility, plus the stability of recorded signals across a single substrate and between different substrates. The morphology of these SERS-active substrates was visualized by the scanning electron microscopy (SEM) technique and is presented in Supplementary Materials (Fig. S1).

The selected interleukin antibodies anti-IL6, anti-IL8, and anti-IL-18 were immobilized onto the Ag-Au SERS-active substrate described above via 6-amino-1-hexanethiols layers using EDC/NHS chemistry (see Supplementary Materials). In the next step, the SERS activities of Raman reporters, i.e. FC, *p*-MBA, and DTNB-labeled immune Au nanoparticles were examined.

Figure S5 presents an example of the successful binding of DTNB and IL-8 antibodies to the Au nanoparticles using UV-vis measurements. As shown in Fig. S5a, the as–received gold nanoparticles solution has a strong extinction maximum at 609 nm. This wavelength is indicative of individual nanoparticles averaging approximately 70 nm in diameter. The spectrum of AuNPs red-shifted a little after Raman reporter coating (Fig. S5b), since the LSPR band of AuNPs is very sensitive to the refractive index of the surrounding medium. The red shift of the surface plasmon resonance peak^22^ demonstrates the successful binding of DTNB to the gold nanoparticles. After modification with IL-8 antibodies one can observe a large decrease in the strength of this band and a red-shift from 609 to 637 nm, which indicates that surface residual vacancies of DTNB-labelled AuNPs are occupied by anti-IL-8 antibodies (Fig. S5c). Similar results have been observed and detected by the ATR/FTIR technique^23^. Additionally, according to the literature^24^ the broadening and the shift to longer wavelength may also indicate aggregation.

**Figure 2:**
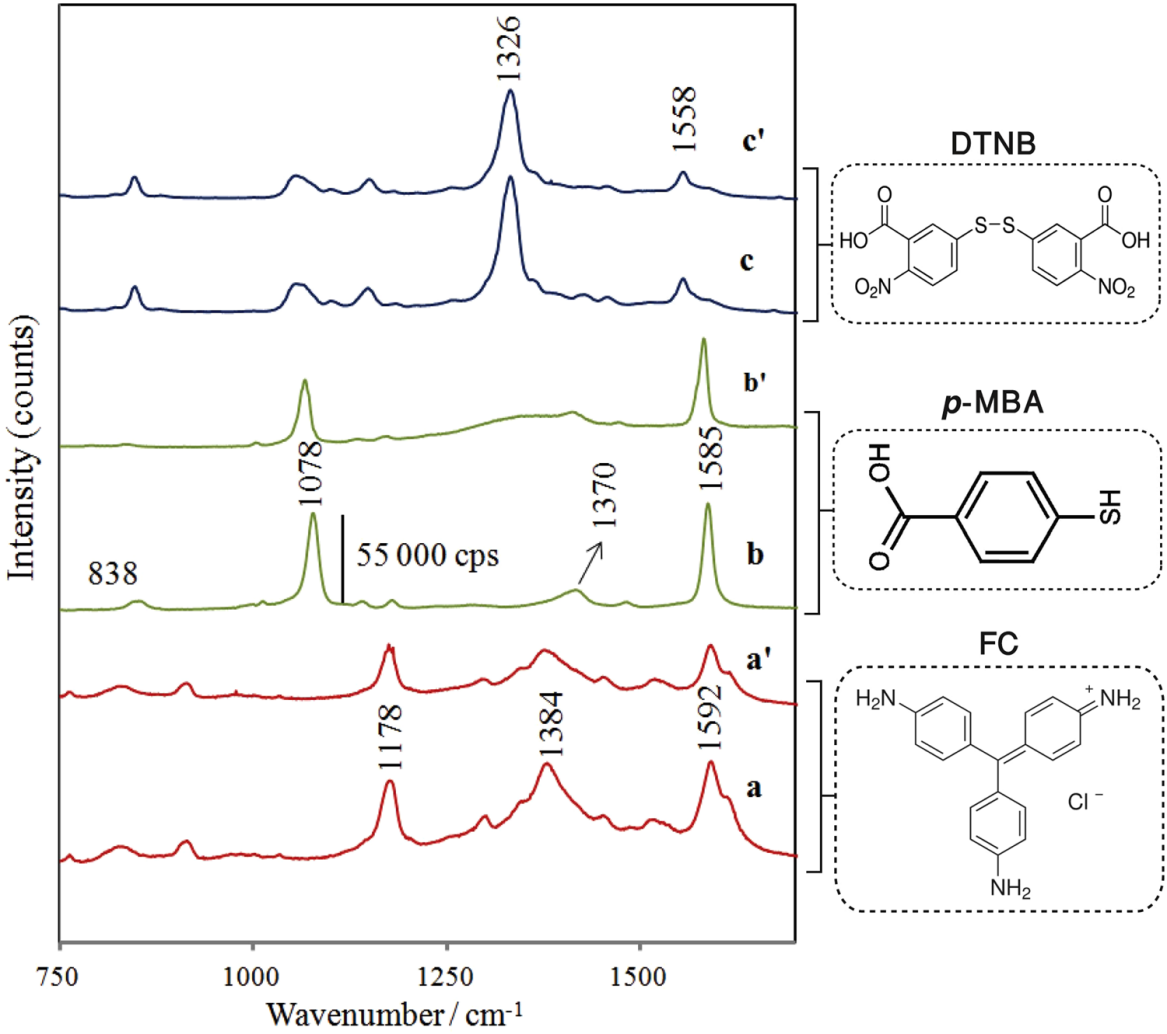
(**a,b,c**) SERS spectra of FC, *p*-MBA, and DTNB adsorbed onto AuNPs (Raman reporter-AuNPs), and (a’,b’,c’) SERS spectra of FC, *p*-MBA, and DTNB–labeled immune AuNPs (antibody-Raman reporter-AuNPs), respectively.

The SERS labels (Raman reporters) with distinct spectroscopic peaks are needed to achieve simultaneous and multiplexed biomarker detection. The Raman reporter molecules have been strategically designed to immobilize them on a thiolate layer on a gold nanoparticle, in order to provide a unique and strong Raman spectrum and to covalently bind the antibody via a terminal amino (FC) or carboxy (*p*-MBA, DTNB) group. Normalized SERS spectra and the corresponding molecular structures from three Raman reporters: FC, *p*-MBA, and DTNB were obtained and are shown in Fig. 2. The SERS spectrum of each type of label can be easily distinguished because the most intense peaks of each Raman reporter molecule are separated by at least 30 cm^−1^.

The SERS spectrum of FC (Fig. 2a) is very intense and dominated by bands at 1178, 1384, and 1592 cm^−1^ due to ring C–H bending modes, N–phenyl stretching, and ring C–C stretching modes^25^, respectively. SERS spectra from other types of SERS labels were characterized accordingly. The SERS spectrum of *p*-MBA (Fig. 2b) is dominated by the bands around 1078 and 1585 cm^−1^ which are assigned to ν_8a_ and ν_12_ aromatic ring vibrations, respectively^26^. The weaker bands which appeared at about 1370–1380 cm^−1^ correspond to ν_s_(COO^−^), together with a band at 838 cm^−1^ due to ν(COO^−^). The relatively intensive peak at 713 cm^−1^ is assigned to the out-of-plane ν(CCC) vibration^27^ of the aromatic ring, indicating that *p*-MBA molecules with COO^−^ groups are more or less flat oriented on the Au surface. The spectral features of the last applied Raman reporter DTNB after the Au staining enhancement is presented in Fig. 2c. The strongest SERS bands that appear at 1326 cm^−1^ and 1558 cm^−1^ are assigned to the symmetric nitro stretch ν_s_(NO_2_) mode and the aromatic ring stretching mode, respectively^28^. The peaks at 1139 and 1048 cm^−1^ are attributed to CH_3_ rocking, C-N stretching, and C-N bending^29^.

Similarly, strong characteristic SERS signals of *p*-MBA, FC, and DTNB (Fig. 2a’–c’) were obtained for each Raman reporter-labeled immuno-Au-nanoparticle (after immobilizing the anti IL-6, anti IL-8, and anti IL-18 antibodies), which indicates that these labels can work as sensitive Raman reporters for SERS immunoassay.

### Detecting SERS-based IL-6, IL-8, and IL-18 interleukins

Immunoassays may come in many different formats and variations, but usually run in multiple steps with reagents being added and washed away or separated at different points in the assay^30, 31^. Such a strategy may cause problems which complicate the interpretation of data and may lead to wrong diagnoses. In the proposed SERS-based immunoassay we solve this difficulty via applying the designed microfluidic device. The signals could be collected from the same point on the SERS-assay (Fig. 1), which additionally enhances the reproducibility of the SERS-based immunoassay method. Moreover, the incorporation of SERS-active nanostructures into a microfluidic device offers a significantly larger active surface for immune reactions and hence improved performance of the immunoassay.

Our experiments were performed in two ways to demonstrate the potential of our assay for detecting particular and multiplexed IL-6, IL-8, and IL-18 interleukins in human blood plasma samples. In the first approach (parallel detection), individual SERS platforms in detection area DA1-3 were functionalized with antibodies against selected interleukins – anti-IL6, anti-IL8, and anti-IL18, respectively (Fig. S6).

**Figure 3:**
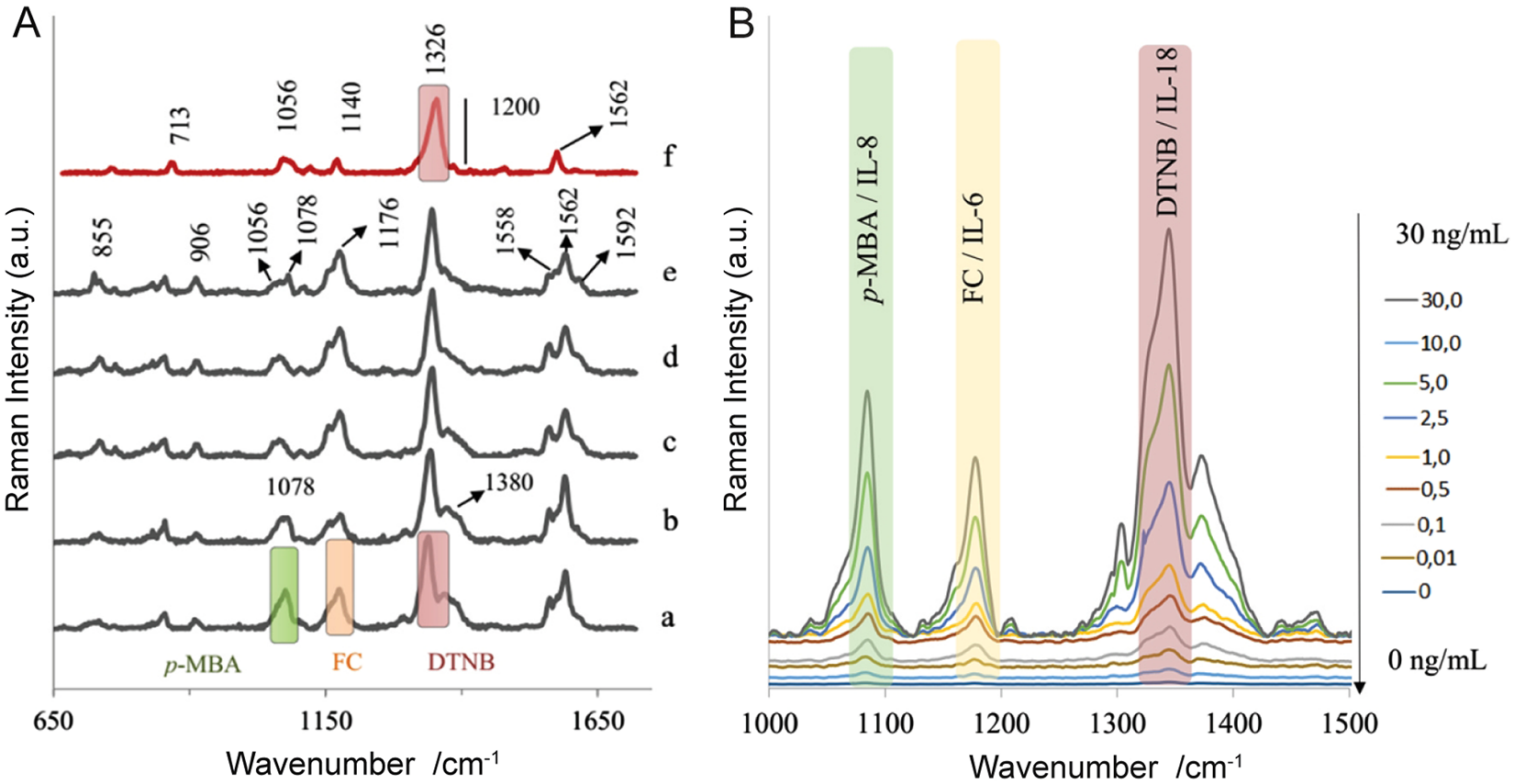
(**A**) SERS responses in the DA-1 chamber of microfluidic immunoassay during detection of IL-18 in human blood plasma sample according to the subsequent steps of parallel approach: (a) after the mixture of equal amounts of three kinds of Raman reporter-labeled Au nanoparticles was delivered and incubated (5 min) onto the anti-IL18 modified Ag-Au SERS-active surface; (b–f) after subsequent washing by PBS buffer solution. (**B**) Typical SERS responses for increasing concentration of target IL-6, IL-8, and IL-18 interleukins in blood plasma during a simultaneous multiplexed approach.

Figure 3A shows the SERS signals recorded in the DA-1 chamber of microfluidic immunoassay in the parallel configuration of the chip (see Fig. 1D and Fig. 6SB), where a mixture of three kinds of Raman reporter-labeled Au nanoparticles (anti-IL6/AuNPs-FC; anti-IL8/AuNPs-p-MBA; anti-IL18/AuNPs-DTNB) (injected via inlet O_2_) and target interleukins IL-6, IL-8, and IL-18 in human blood plasma (injected via inlets X_2_, Y, and Z_2_, respectively) were mixed and then delivered via tube H1 to the anti-IL18 modified Ag-Au SERS-active surface (DA-1). As one can see in Fig. 3A(a) the SERS spectrum revealed strong characteristic peaks for DTNB at 1326 cm^−1^, *p*-MBA at 1078 cm^−1^, and FC at 1176 cm^−1^. As the wash time increased, the SERS spectral response showed decreasing intensity of the bands of *p*-MBA at 1078 cm^−1^ and FC at 1176 cm^−1^ (Fig. 3A(b–e)); finally, after 2 minutes, these bands practically disappeared (Fig. 3Af). At the same time, it was also observed that there were no remarkable changes in the intensity of the bands corresponding to DTNB during the washing steps. This shows the high specificity of the developed SERS immunoassay and indicates its potential for IL-18 detection even in complex biological fluids. The same protocol was adopted and similar experiments performed to investigate the ability of the SERS immunoassay for the detection of IL-6 and IL-8 interleukins from the blood plasma samples.

A control experiment has also been performed to verify the selectivity of the immunological recognitions. Figure S7 presents the typical SERS spectra recorded in response to different combinations of target interleukins, including also the unspecific Akt blocking peptide for all studied antibodies employed in the same immunoassay protocol, as described in Fig. 1. The Akt blocking peptide specifically binds only the Akt (pan) Rabitt mAb antibody. As can be seen in Fig. 3a, after the addition of Akt blocking peptide, there were no remarkable bands (except extremely weak ones, e.g. 1382 cm^−1^) originating from any of the Raman reporter molecules, indicating antigen-antibody interactions. Some weak vibrational transitions that appeared at 762, 1003, 1021, 1452 and 1615 cm^−1^ may arise from human blood plasma components (proteins, carbohydrates, lipoproteins or metabolites).

When the mixture of three kinds of Raman reporter-labeled Au nanoparticles (anti-IL6/AuNPs-FC; anti-IL8/AuNPs-*p* -MBA; anti-IL18/AuNPs-DTNB) was injected and then incubated with an individual interleukin IL-6 and IL-8 in blood plasma samples in each detection area chamber (Fig. 1, DA 2 and 3), strong SERS signals originating from the particular Raman reporter molecules (Fig. 3B(c–d)) appeared. The characteristic fingerprint spectrum of each Raman reporter in fact provides a unique code for the particular antibody and for the target interleukins.

These experiments suggest that this approach is potentially suitable for detecting multiplexed interleukins from blood plasma samples. Therefore, in the second approach (simultaneous multiplex detection), the individual SERS-active platforms were functionalized with three, anti-IL6, anti-IL8, and anti-IL18 antibodies to permit simultaneous detection of three target interleukins. Blood plasma samples with intended concentrations of IL-6, IL-8, and IL-18 interleukins and the three kinds of Raman reporter-labeled Au nanoparticles (anti-IL6/AuNPs-FC; anti-IL8/AuNPs-p -MBA; anti-IL18/AuNPs-DTNB) were applied to the detection area chambers (Fig. S6). It should be highlighted that at each of the analysed concentrations, equal concentrations of the three target interleukins were added to blood samples. Figure 3B illustrates the simultaneous detection of three target interleukins in blood plasma samples. As can be seen, the mixture of three interleukins (IL-6, IL-8, IL-18) in human blood plasma samples was correspondingly encoded by distinct Raman reporters: FC, *p*-MBA, and DTNB, thus enabling the simultaneous and multiplexed detection of three biomarkers. Specifically, IL-6 interleukin is encoded using the characteristic peak for FC at 1178 cm^−1^, IL-8 is defined by the characteristic peak for *p*-MBA at 1078 cm^−1^, and IL-18 interleukin is determined by the characteristic peak for DTNB at 1326 cm^−1^ (see Figs 2 and 3). These results demonstrated the capability of our SERS-based immunoassay for multiplexed detection of three interleukins even in complex biological fluids.

### Quantitative analysis

**Figure 4:**
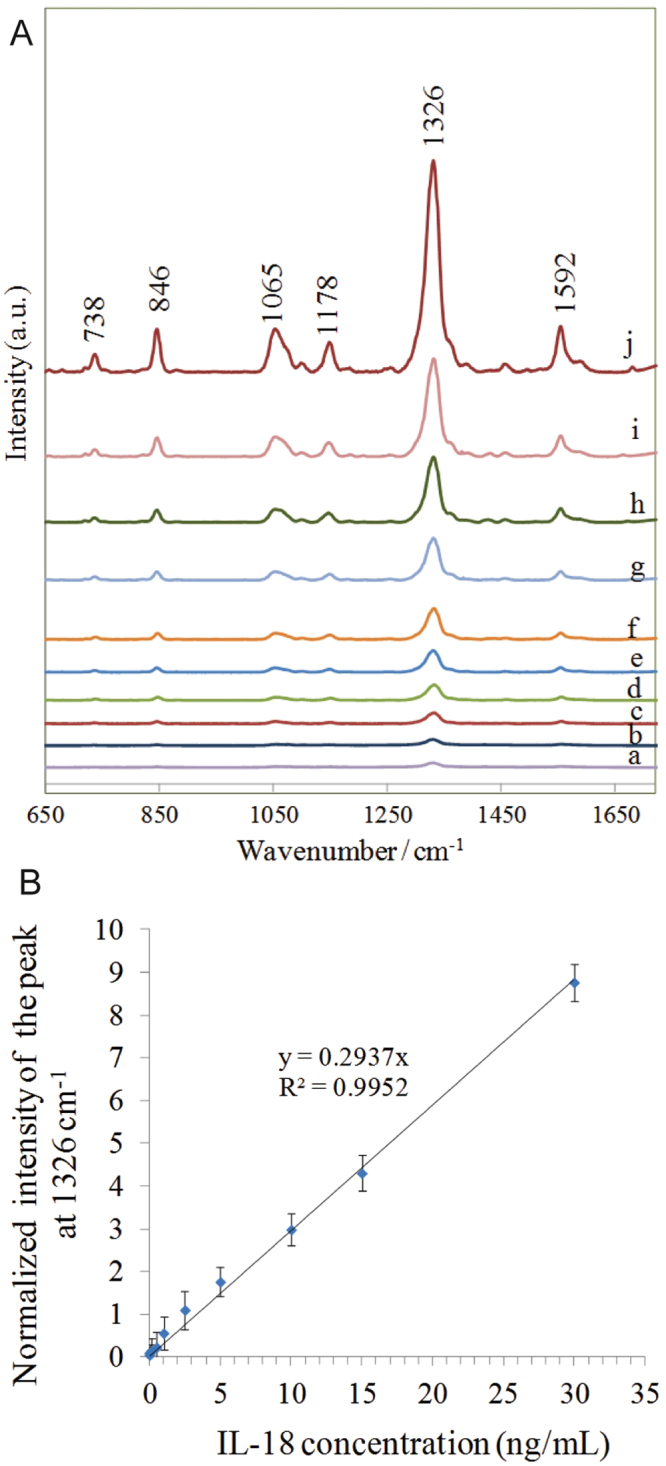
(**A**) SERS spectra obtained for increasing concentration of IL-18: (a) 0.0; (b) 0.005; (c) 0.01; 0.1; (e) 0.5; (f) 1.0; (g) 2.5; (h) 5.0; (i) 10.0; (j) 30.0 ng·ml^−1^ in blood plasma. (**B**) The relationship between the intensity of the marker band at 1326 cm^−1^ versus the concentration of IL-18 in the range from 0 ng·ml^−1^ to 30 ng·ml^−1^.

The capability of the developed SERS-based immunoassay for quantitative analysis of three studied interleukins in parallel and simultaneous multiplexing approaches was tested by measuring the SERS responses for varying concentrations of these target interleukins: IL-6, IL-8, and IL-18 in blood plasma samples. Figure 4 presents the results of the parallel SERS detection of IL-18. A dilution series of IL-18 in human blood plasma in the range of 0–30 ng·ml^−1^ were prepared. The concentration range was chosen to cover clinically relevant concentrations of interleukin IL-18. Figure 4a–j present the SERS spectra for selected concentrations after completion of the immunoassay protocol outlined above. Various concentrations of the target interleukins were applied using a microfluidic system. In order to increase the density of immunocomplexes, the SERS spectra were measured under a steady state condition in the reaction chamber. We checked that under continuous flow conditions, the intensities of SERS signals were too low to perform a quantitative analysis. Therefore, in our microfluidic chip we combined the continuous flow of reagents with the static flow (the incubation step).

It should be highlighted that in order to achieve reproducible data, the confocal Raman mapping mode was employed to solve problems with the non-uniform spatial distribution or aggregation of SERS nanoparticles onto the SERS substrate. The blank spectrum (Fig. 4a) was recorded for the blank blood plasma sample (from a healthy subject) without adding IL-18 antigen. Without adding the complementary antigens, the Raman reporter-labeled immuno-Au-nanoparticles should be easily removed from the substrate by the washing process. Additionally, to further demonstrate the specificity of immunocapture, control experiments were also performed in a PBS buffer solution without antigens (Fig. S8). As can be seen, negligible nonspecific adsorption was observed in all cases – in the blank blood plasma and the PBS samples. This indicates that, in spite of the rigorous washing process, a small number of DTNB-labeled immuno-Au nanoparticles may adsorb onto the SERS-active substrate without any immune recognition. Nevertheless, the difference in the intensity of the SERS signals obtained under specific and unspecific recognition regimes clearly demonstrates the high specificity of our immunoassay.

Figure 4A shows SERS spectra obtained for each tested concentration of IL-18 interleukins. Figure 4B demonstrates the relationship between the intensity of the marker band at 1326 cm^−1^ versus the concentration of target interleukin over the whole tested concentration range. Figure 4B illustrates a calibration curve obtained by plotting the intensity of the SERS signal of DTNB (Raman reporter) marker band at 1326 cm^−1^ versus the concentration of the antigen in the range from 0 ng·ml^−1^ to 30 ng·ml^−1^.

The error bars indicate the standard deviations from ten measurements of different spots for each concentration. In the linear region the calibration curve was fitted as y = 0.2937x and the coefficient of determination (R^2^) was 0.99. For the linear calibration curve, it was assumed that the SERS intensity at 1326 cm^−1^ (y) is linearly related to the concentration of the target interleukin (x).

In addition, a low detection limit (LOD) was estimated using the signal-to-noise (S/N) method^32^. A signal-to-noise ratio of three is generally accepted for estimating LOD. Based on these data, the detection limit was calculated to be 4.2 pg·ml^−1^, corresponding to clinical concentrations of IL-18 in body fluids. The current detection limit for IL-18 using conventional ELISA test is about 15.6 pg·ml^−1^ ^33^. Blood plasma IL-18 levels were significantly elevated in diabetic patients and range from 2.05–14.8 ng·ml^−1^ as compared with control subject (1.2–1.6 ng·ml^−1^)^34^.

Additionally, the reproducibility of the presented SERS immunoassay towards detecting IL-18 was also investigated. Figure S9 shows 15 individual readings from 1 to 15 randomly selected spots for three different SERS surfaces immersed in human blood plasma with different concentrations of IL-18 interleukin (0.5, 1.5, and 30.0 ng·ml^−1^). To get a statistically valid result, the marker band of Raman reporter at 1326 cm^−1^ was chosen to calculate the relative standard deviation (RSD). The corresponding relative standard deviations were 8.5%, 7.7%, and 7.0%, respectively. The relative average standard deviation (RSD) of this method is less than 8.5%, which is comparable to that of conventional ELISA assays.

The excellent reproducibility is due to applying the microfluidic device that enables performing and monitoring the subsequent and controlled immunoreactions. Moreover, the proposed architecture allows for the collection of SERS signals from one spot on a SERS-active platform during the whole process of detection.

Figure S10 (ESI) shows typical SERS signals of the SERS-based immunoassay for interleukin IL-8. We can also observe that SERS-band intensities increase steadily with increasing concentrations of interleukin IL-8 in injected plasma blood samples. The quantitative analysis was performed by measuring the SERS intensity of the p-MBA marker band at 1078 cm^−1^ versus the concentration of interleukin in the range from 0 ng·ml^−1^ to 30 ng·ml^−1^. The detection limit was calculated to be 6.5 pg·ml^−1^ in terms of the rules applied in the signal-to-noise method^32^. The average IL-8 plasma concentration in the control group was 14.8 ng·ml^−1^ and significantly increased in patients with acute respiratory distress syndrome to 84 ng·ml^−1^ ^35^.

The relative average standard deviation (RSD) of *p*-MBA marker band at 1078 cm^−1^ was 6.2%. Figure S11 shows the reproducibility SERS spectra of *p*-MBA adsorbed onto the SERS platform from 2 ng·ml^−1^ solution of *p*-MBA in human blood plasma. To compare, the lower limit of sensitivity for IL-8 detection using ELISA assay was found to be 64 pg·ml^−1^ with an inter-assay variability of 15–29% and an intra-assay variability of 12%^36^.

Figure S12 presents the SERS responses in quantitative analysis of IL-6 interleukin in the developed multiplexed immunoassay. The intensity of the fingerprint spectra of Raman reporter FC encoding interleukin IL-6 was also found to increase with increasing concentrations of IL-6 in injected blood plasma samples. The SERS band at 1176 cm^−1^ revealed a linear correlation to IL-6 concentrations through the range from 0 ng·ml^−1^ to 30 ng·ml^−1^ with a detection limit of 2.3 pg·ml^−1^. IL-6 serum concentrations may increase up to 229 ng·ml^−1^ for patients with active rheumatoid arthritis^37^.

The relative average standard deviation (RSD) of the FC marker band at 1176 cm^−1^ was 6.5% from three repetitive assays of 1.0 ng·ml^−1^, 2 ng·ml^−1^, and 20 ng·ml^−1^ of IL-6. Figure S13 shows the reproducible SERS spectra of FC adsorbed onto the SERS platform from a 2 ng·ml^−1^ solution of FC in human blood plasma.

**Figure 5:**
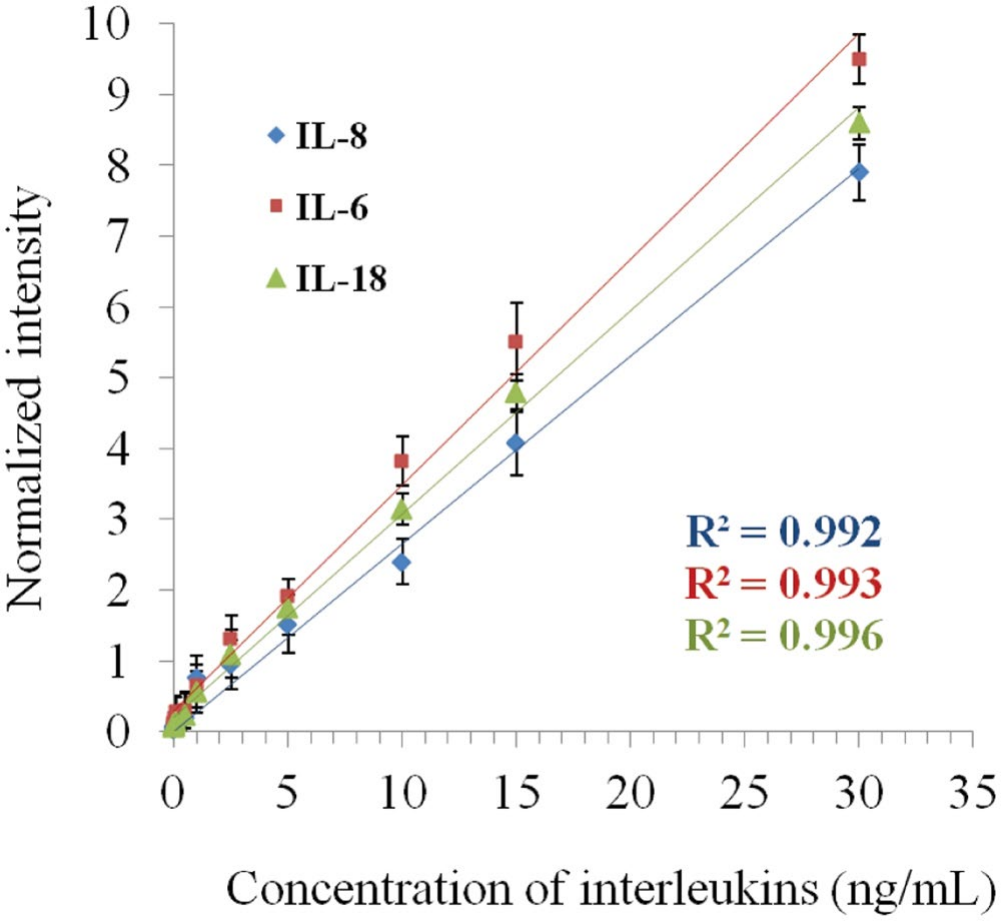
The concentration – intensity calibration curves obtained for simultaneous multiplexed detection of three IL-6, IL-8, and IL-18 interleukins from blood plasma samples. Each SERS spectrum was averaged from 20 measurements at different places across the SERS surface using the mapping mode. The error bars indicate the standard deviations from twenty measurements at different spots for each concentration.

It is evident (Fig. 3B) that our assay demonstrates the multiplexing capability for simultaneous detection of three tested interleukins. A linear increase in SERS intensities was observed with an increase in concentrations across all three tested interleukins. The characteristic Raman peaks for *p*-MBA at 1078 cm^−1^, FC at 1178 cm^−1^, and DTNB at 1326 cm^−1^ encoded IL-6, IL-8, and IL-18 interleukins were used to monitor the quantity of each target interleukin. Figure 5 presents the concentration - intensity response curves for simultaneous detection of three interleukins from blood serum samples. Figure S14 presents the same results in a semi-log plot for better visibility.

From these calibration curves the low detection limits (LODs) of IL-6, IL-8, and IL-18 were estimated to be 3.8 pg·ml^−1^, 7.5 pg·ml^−1^, and 5.2 pg·ml^−1^, respectively. This means that the sensitivity of multiplexed simultaneous detection assay for triple interleukins provides, as in the case of the parallel detection approach (with LODs at 2.3 pg·ml^−1^, 6.5 pg·ml^−1^, and 4.12 pg·ml^−1^ for IL-6, IL-8, and IL-18, respectively), a high detection throughput within the clinically relevant range but with lower sample con**s**umption compared with a parallel assay. The current detection limit for IL-6, IL-8, and IL-18 using ELISA is about 4.0 pg·ml^−1^ ^38^, 64.5 pg·ml^−1^ ^36^, and 15.6 pg·ml^−1^ ^33^, respectively, which does not always meet the requirements of clinical diagnosis.

These data reveal that the newly developed SERS-based immunoassay offers a new recognition bioplatform for quantitative and simultaneous analysis of three interleukins: IL-6, IL-8, and IL-18 in body fluids with desired sensitivity and reproducibility.

### PCA analysis

To realize identification of Raman reporter molecules in unknown mixtures or in the presence of additional analyte molecules (for example, in complex biological samples) beyond the visual inspection of individual spectra, multivariate methods are advantageous. Principal components analysis (PCA) is a useful statistical technique that considers not only the most intense characteristic bands but the whole spectral fingerprint for finding patterns in data of high dimension, in particular in multiplex detection approaches, especially where fast read-out is required. The spectral differences in data sets comprising 160 spectra obtained for multiplexed simultaneous detection of three tested interleukins from serum blood samples were analyzed by PCA. The main information obtained from the PCA is explained by the two first principal components PC1 and PC2 that cover 97% of the total variance in the data. Figure S15 shows three clusters corresponding to three interleukins IL-6, IL-8, and IL-18, specifically encoded by particular Raman reporters FC, *p*-MBA, and DTNB, respectively. The statistical data show excellent segregation of the obtained data and indicate the possibility to use PCA analysis in the SERS-immunoassay for simultaneous and sensitive determination of all three tested immune markers in human blood samples. The differences among the SERS spectra recorded during multiplexed analysis of interleukins in blood plasma samples are clearly illustrated by the loadings plots presented in Fig. 5B. The loadings of the PCs provide information on the variables (wavenumber of the spectrum) that are important for group separation. Figure 5B displays the loadings plots of PC1 and PC2 for the whole wavenumber region 500–1600 cm^−1^. By analyzing these plots, one can indicate the most important diagnostic variables in the analyzed data set. On the basis of loadings plots from PC-1, the most important variation among these three interleukins IL-6, IL-8, and IL-18, were found to be associated with the bands at 1179, 1078, 1326, and 1593 cm^−1^ corresponding to the characteristic marker bands of Raman reporters. These findings are consistent with those empirically observed in the SERS spectra shown in Fig. 2. Importantly, the differences between samples belonging to the same group of classification (one type of interleukin) are not diagnostically significant. These results clearly demonstrate the potential of PCA-based SERS immunoassay method for multiplexed detection of immune markers in clinical analysis.

## Conclusions

To the best of our knowledge, this is the first report on the construction of SERS-based immunoassay in a microfluidic device for simultaneous and multiplex analysis of three interleukins IL-6, IL-8, and IL-18, in human blood plasma samples. Our approach offers a comprehensive multiplexing capability, with high sensitivity and specificity owing to selective formation of conjugated antigen-antibody complexes. These complexes are encoded by Raman reporter molecules, which serve as extrinsic labels for each type of interleukin. The presence of a specific interleukin is determined by the characteristic marker bands of Raman reporter molecules.

Crucial constructs of the developed SERS immunoassay include: (i) a SERS-active surface that supports a SERS enhancement factor that is substantial enough to detect interleukins at physiologically relevant concentrations, (ii) an appropriately designed Raman reporter-labeled AuNPs, (iii) the microfluidic device for multiplexed analysis *in situ*, and (iv) a multivariate statistical method (PCA) to resolve interleukins signals within mixtures.

The estimated values of low detection limits and average standard deviations of the marker bands for all interleukins IL-6, IL-8, and IL-18 exhibit high sensitivity, reflecting clinical relevant interleukin concentrations and the excellent reproducibility desirable for analytical analysis. Our developed SERS-based immunoassay improves the detection limits of these three interleukins in comparison to conventional ELISA tests and demonstrates the potential for quantitative detection of interleukins in complex biological fluids.

In the future our strategy can be extended to the detection of interleukins in blood plasma from clinical samples. The successful completion of the presented studies are a good starting point for designing practical sensors for immune markers applicable in clinical practice.

## Electronic supplementary material

Dataset 1

## References

[1] Wang YL, Salehi M, Schutz M, Rudi K, Schlucker S (2013). Microspectroscopic SERS detection of interleukin-6 with rationally designed gold/silver nanoshells. Analyst.

[2] Wang YL, Salehi M, Schutz M, Schlucker S (2014). Femtogram detection of cytokines in a direct dot-blot assay using SERS microspectroscopy and hydrophilically stabilized Au-Ag nanoshells. Chem. Commun..

[3] Ledur A, Fitting C, David B, Hamberger C, Cavaillon JM (1995). Variable estimates of cytokine levels produced by commercial ELISA kits: results using international cytokine standards. J. Immunol. Methods.

[4] Miresluis AR, Das RG, Thorpe R (1995). The international standard for macrophage colony stimulating factor (M-CSF). Evaluation in an international collaborative study. J. Immunol. Methods.

[5] Miresluis AR, Das RG, Thorpe R (1995). The international standard for macrophage colony stimulating factor (M-CSF). Evaluation in an international collaborative study. J. Immunol. Methods.

[6] Scheller J, Rose-John S (2006). Interleukin-6 and its receptor: from bench to bedside. Med. Microbiol. Immunol..

[7] Cho YA, Sung M-K, Yeon J-Y, Ro J, Kim J (2013). Prognostic role of interleukin-6, interleukin-8, and leptin levels according to breast cancer subtype. Cancer Research and Treatment: Official Journal of Korean Cancer Association.

[8] Chang CH (2013). Circulating interleukin-6 level is a prognostic marker for survival in advanced nonsmall cell lung cancer patients treated with chemotherapy. International journal of cancer.

[9] Yokoe T, lino Y, Morishita Y (2000). Trends of IL-6 and IL-8 levels in patients with recurrent breast cancer: preliminary report. Breast Cancer.

[10] Combs ZA (2013). Aptamer-assisted assembly of gold nanoframe dimers. Part. Part. Syst. Charact..

[11] Ko H, Chang S, Tsukruk VV (2009). Porous substrates for label-free molecular level detection of nonresonant organic molecules. Acs Nano.

[12] Ko H, Singamaneni S, Tsukruk VV (2008). Nanostructured surfaces and assemblies as SERS media. Small.

[13] Kneipp K (1998). Detection and identification of a single DNA base molecule using surface-enhanced Raman scattering (SERS). Phys. Rev. E.

[14] Stokes RJ (2008). Surface-enhanced Raman scattering spectroscopy as a sensitive and selective technique for the detection of folic acid in water and human serum. Appl. Spectrosc..

[15] Faulds K, Smith WE, Graham D, Lacey RJ (2002). Assessment of silver and gold substrates for the detection of amphetamine sulfate by surface enhanced Raman scattering (SERS). Analyst.

[16] Alexander TA, Le DM (2007). Characterization of a commercialized SERS-active substrate and its application to the identification of intact Bacillus endospores. Appl. Opt..

[17] Sivanesan A (2014). Nanostructured silver-gold bimetallic SERS substrates for selective identification of bacteria in human blood. Analyst.

[18] Patent application: P-406026 (2013).

[19] Mabbott S, Eckmann A, Casiraghi C, Goodacre R (2013). 2p or not 2p: tuppence-based SERS for the detection of illicit materials. Analyst.

[20] Kaminska A, Kowalska A, Albrycht P, Witkowska E, Waluk J (2016). ABO blood groups’ antigen-antibody interactions studied using SERS spectroscopy: towards blood typing. Anal. Methods.

[21] Szymborski T, Witkowska E, Adamkiewicz W, Waluk J, Kaminska A (2014). Electrospun polymer mat as a SERS platform for the immobilization and detection of bacteria from fluids. Analyst.

[22] Mie G (1908). Beiträge zur Optik trüber Medien, speziell kolloidaler Metallösungen. Annalen der Physik.

[23] Song CY, Wang ZY, Yang J, Zhang RH, Cui YP (2011). Effects of solid substrate on the SERS-based immunoassay: a comparative study. J. Raman Spectrosc..

[24] Ni J, Lipert RJ, Dawson GB, Porter MD (1999). Immunoassay readout method using extrinsic Raman labels adsorbed on immunogold colloids. Anal. Chem..

[25] Moskovits M, Suh JS (1984). Surface selection-rules for surface-enhanced Raman-spectroscopy: calculations and application to the surface-enhanced Raman spectrum of phthalazine on silver. J. Phys. Chem..

[26] Park HK, Lee SB, Kim K, Kim MS (1990). Surface-enhanced Raman scattering of p-aminobenzoic acid at Ag electrode. J. Phys. Chem..

[27] Lee SB, Kim K, Kim MS (1991). Surface-enhanced Raman scattering of o-mercaptobenzoic acid in silver sol. J. Raman Spectrosc..

[28] Guven B, Basaran-Akgul N, Temur E, Tamer U, Boyaci IH (2011). SERS-based sandwich immunoassay using antibody coated magnetic nanoparticles for Escherichia coli enumeration. Analyst.

[29] Grubisha DS, Lipert RJ, Park HY, Driskell J, Porter MD (2003). Femtomolar detection of prostate-specific antigen: an immunoassay based on surface-enhanced Raman scattering and immunogold labels. Anal. Chem..

[30] Wang Y, Tang LJ, Jiang JH (2013). Surface-enhanced Raman spectroscopy-based, homogeneous, multiplexed immunoassay with antibody-fragments-decorated gold nanoparticles. Anal. Chem..

[31] Han XX (2009). Protein-mediated sandwich strategy for surface-enhanced Raman scattering: application to versatile protein detection. Anal. Chem..

[32] Shrivastava A, Gupta V (2011). Methods for the determination of limit of detection and limit of quantitation of the analytical methods. Chronicles of Young Scientists.

[33] Osaki T (1999). Potent antitumor effects mediated by local expression of the mature form of the interferon-gamma inducing factor, interleukin-18 (IL-18). Gene therapy.

[34] Janiak A, Leśniowski B, Jasińska A, Pietruczuk M, Małecka-Panas E (2015). Interleukin 18 as an early marker or prognostic factor in acute pancreatitis. Przegla̜d Gastroenterologiczny.

[35] Kozlowski L, Zakrzewska I, Tokajuk P, Wojtukiewicz MZ (2003). Concentration of interleukin-6 (IL-6), interleukin-8 (IL-8) and interleukin-10 (IL-10) in blood serum of breast cancer patients. Roczniki Akademii Medycznej w Bialymstoku (1995).

[36] DeForge LE, Remick DG (1991). Sandwich ELISA for detection of picogram quantities of interleukin-8. Immunological investigations.

[37] Robak T, Gladalska A, Stepien H, Robak E (1998). Serum levels of interleukin-6 type cytokines and soluble interleukin-6 receptor in patients with rheumatoid arthritis. Mediators of inflammation.

[38] https://www.thermofisher.com.

