# Supplementary material for: SERS-based Immunoassay in a Microfluidic System for the Multiplexed Recognition of Interleukins from Blood Plasma: Towards Picogram Detection
Source: Sci Rep. 2017 Sep 6;7:10656. doi: 10.1038/s41598-017-11152-w (PMC5587571; doi:10.1038/s41598-017-11152-w)
Supplement: Supplementary file 1 — Dataset 1 [file 41598_2017_11152_MOESM1_ESM.doc]

**Supplementary Materials**

SERS-based Immunoassay in a Microfluidic System for the Multiplexed Recognition of Interleukins from Blood Plasma: Towards Picogram Detection

Agnieszka Kamińska^a*^, Katarzyna Winkler^a^, Aneta Kowalska^a^, Evelin Witkowska^a^, Tomasz Szymborski^a^, Anna Janeczek^a^ and Jacek Waluk^a,b^

^a^Institute of Physical Chemistry, Polish Academy of Sciences, Kasprzaka 44/52, 01-224 Warsaw, Poland

^b^Faculty of Mathematics and Natural Sciences, College of Science, Cardinal Stefan Wyszyński University, Dewajtis 5, 01-815 Warsaw, Poland

1. **Capturing substrate characterization.**

| 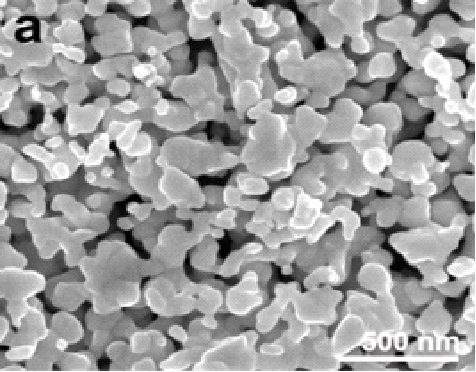  A |
| --- |
| 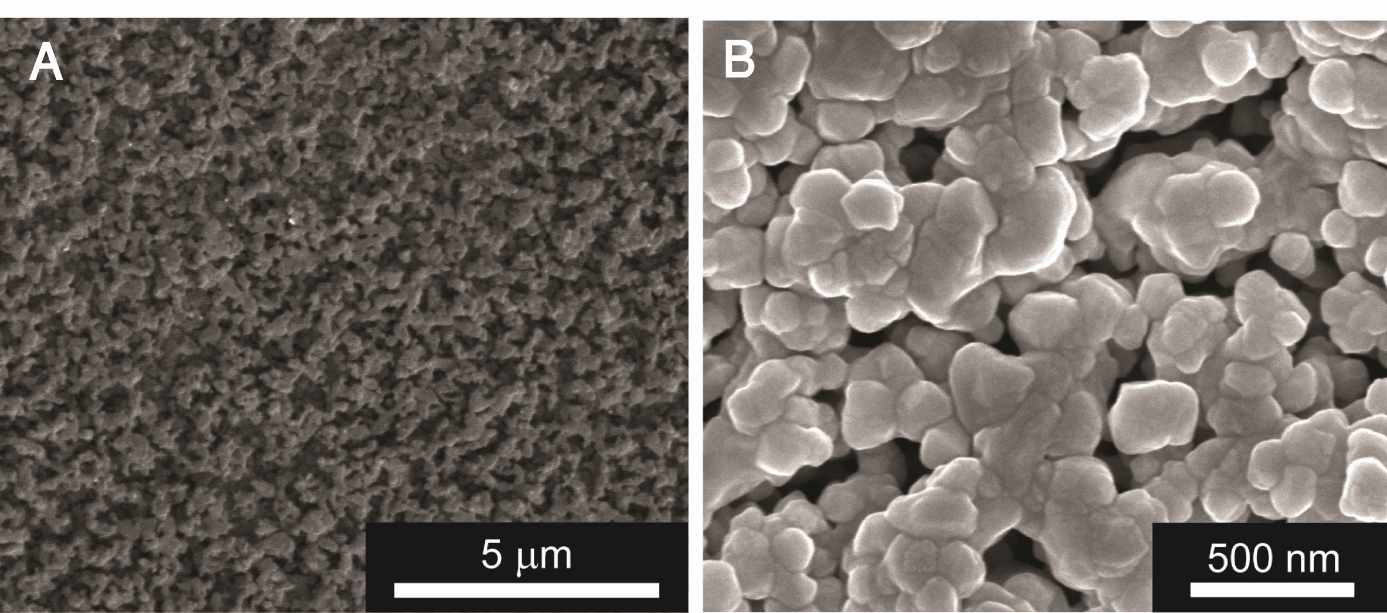  B  C |
| **Fig. S1.** The SEM images of (A) roughened Ag and (B), (C) Ag-Au hybrid surfaces at different magnifications. |

Some of the latest works successfully employed bimetallic nanoparticles as SERS substrates with higher signal enhancement and biocompatibility than the monometallic nanoparticles. By applying the Au-Ag hybrid we can combine high chemical stability of Au with very high Raman scattering enhancement for Ag.

The morphology of the silver-gold bimetallic surfaces was studied by SEM imaging. The results are shown in Fig. S1. The SEM image of rough silver (Fig.S1A) reveals a nanoporous structure with the grain size ranging from 30 to 200 nm. The SEM image of silver-gold bimetallic surface (Fig. 1c) shows, in contrast to the rough silver surface, a more uniform nanostructure with less porosity and increased grain size. Viewed over a wider scanning area (Fig. S1B), the substrate is homogeneous. The closer view of the bimetallic surface depicts crystalline structure for the deposited gold layer (Fig. S1C). Additionally, X-ray photoelectron spectroscopy (XPS) –intensity data for Au 4f7/2 and Ag 3d5/2 electrons revealed the percentage of Au and Ag in the bimetallic surface as 7.6 and 87.4, respectively, assuming the same cross-section for bulk and bimetallic surface (Sivanesan et al, 2014). The remaining 5 percent can be attributed to chlorine and oxygen. The estimated low percentage of Au in the bimetallic surface confirms that it forms a thin layer on the Ag base. A more detailed description of the physicochemical properties of the Au-Ag substrate used in this work has been already published in our previous work (Sivanesan et al., 2014).


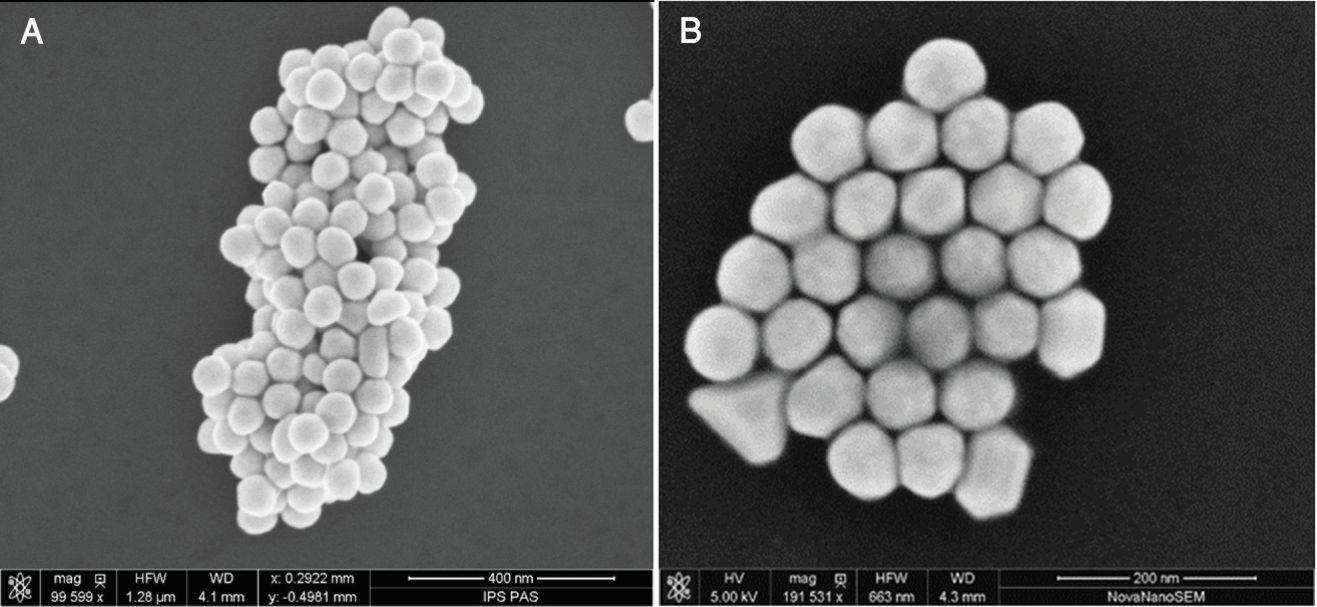


**Fig. S2.** The SEM images of Au nanoparticles at different magnifications (AuNPs@CTAB).

Additionally, the stability of a SERS substrate that determines the range of its practical applications in chemical and biological analysis has been also analysed. The crucial parameters are the stability against oxidation for an extended period of time. Considering the 1072 cm^-1^ band as a reference, the intensity of *p*-MBA was reduced approximately by only 3 % after three months of storing the surface under atmospheric conditions Such high stability and reproducibility enables the quantitative SERS studies of numerous biomolecules and improves the SERS potential in real applications.

1. ***Preparation of the Raman reporter-labeled immuno-Au nanoparticles (anti-IL6 /AuNPs-FC; anti-IL8 /AuNPs-P-MBA; anti-IL16 /AuNPs-DTNB)***

For the multiplex SERS immunoassay, a set of Raman reporters (FC, *p*-PMBA, and DTNB- labeled immune-Au nanoparticles) was synthesized.

- 1. **Synthesis of Au nanoparticles**

Gold nanoparticles of 70 nm in diameter and capped with CTAB (AuNPs@CTAB) were obtained via a two-step seeding protocol^1^. In the first step AuNPs having 15 nm in diameter were synthesized using a Turkevich approach^2^. In this approach, seeds were obtained by addition of HAuCl_4_ × 3H_2_O (49 mg, 0.125 mmol) to 250 mL of boiling water, followed by addition of trisodium citrate dihydrate (125 mg, 0.425 mmol). In the second step, CTAB (2.74 g, 7.5 mmol) was dissolved in 500 mL of water heated to 35 °C. Then, solution of HAuCl_4_ × 3H_2_O in water (2.5 mL, 0.1 M) was added and stirring was continued until the mixture became clear. Next, solution of ascorbic acid (2 mL, 2.5 mM) was injected and, after complete discoloration of the reaction mixture, 6.5 mL of the seed solution was added quickly. The mixture was stirred gently for 1 hour, then centrifuged and carefully decanted. The precipitate was dispersed in 8 mL of CTAB water solution (0.1 M) and left overnight in order to the shape-selective separation of non-spherical particles formed during the growing process. Solution of the spherical AuNPs@CTAB was then carefully collected from above the sediment of the undesired ones. The concentration of the gold atoms in the final solution, determined from the absorption spectrum, was 15.42 mM.

The average diameter of gold nanoparticles was about 70 nm according to the SEM images (Fig. S2 and S3).

In the next step, the Raman reporter-labeled Au nanoparticles were fabricated. Three Raman reporter molecules: FC, *p*-MBA and DTNB were immobilized on the prepared nanoparticles according to the following procedures.


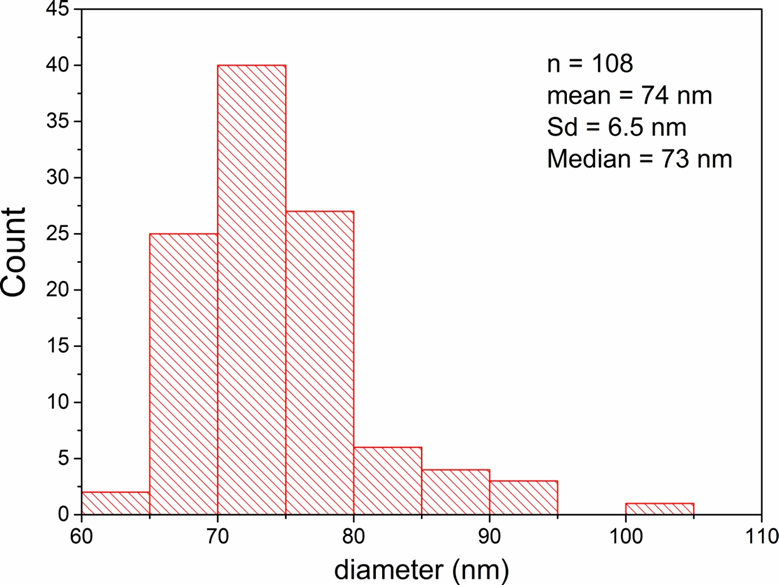


**Fig. S3.** Histogram of diameters of Au nanoparticles.

- 1. ***Synthesis of anti-IL6/AuNPs-FC***

Solution of the AuNPs@CTAB (1 mL) was centrifuged (6000 rpm, 10 min) in order to remove an excess of CTAB. The supernatant liquid was carefully decanted and the precipitate was dissolved in DMF (5 mL). To the solution, water solution of mercaptosuccinic acid (MSA, 100 µL, 0.2 M) was added during stirring. After 12 hours the mixture was cooled to about 0 °C and EDC (10 mg, 0.05 mmol) was added, followed by addition of catalytic amount of NHS and solution of basic fuchsin (500 µL, 200 mM) in DMF (see chapter 3 in Supplementary Materials). MSA-Fuchsin coated AuNPs were collected by centrifugation (5000 rpm, 20 min) and next purified by six-fold dissolution in DMF and centrifugation procedure, and then by ten-fold dissolution in water and centrifugation procedure. The purified AuNPs were dried and suspended in 2.5 mL of water. In the last step, the immune-Raman reporter-labeled Au-nanoparticles (Fig. 1B) were prepared by immobilizing a specific antibody anti-IL6 against studied interleukins IL6 using EDC and NHS coupling reagents. That is, 5 µL of 40 µg/mL anti-IL6 (in a PBS buffer, pH 7.7) were mixed with 10 µL of solution containing Raman reporter-labeled Au nanoparticles and then the coupling reagents (0.2 M EDC/0.05 M NHS; in the volume ratio of 5:1, mixture in deionized water) were added to initiate the conjugation reaction at 4 °C for 4 hours.

- 1. ***Synthesis of anti-IL8/AuNPs-P-MBA***

Solution of the AuNPs@CTAB (0.5 mL) was centrifuged (6000 rpm, 10 min) in order to remove an excess of CTAB. The supernatant liquid was carefully decanted and the precipitate was dissolved in water (0.5 mL). To the solution, solution of *p*-mercaptobenzoic acid (*p*-MBA, 6.7 mg, 0.043 mmol, in 4.2 mL of water and 0.8 mL of MeOH) was added while stirring and the mixture was left overnight. The AuNPs@*p*-MBA were collected by centrifugation (5000 rpm, 10 min) and next purified by sixteen-fold dissolution in water and centrifugation procedure. The purified AuNPs were dried and suspended in 2.5 mL of water. The *p*-MBA-modified Au nanoparticles were then modified with the specific antibody against interleukin IL8 using described above EDC/NHS coupling procedure. A 15 µL of nanoparticle solution and 5 µl of 60 µg/mL anti-IL8 were added to a coupling mixture (0.2 M EDC/0.05 M NHS; in the volume ratio of 5:1, mixture in deionized water) and stored at 4 °C for 4 hours for antibody immobilization.

- 1. ***Synthesis of anti-IL16 /AuNPs-DTNB***

Solution of the AuNPs@CTAB (1 mL) was centrifuged (6000 rpm, 10 min) in order to remove an excess of CTAB. The supernatant liquid was carefully decanted and the precipitate was dissolved in water (1 mL). The solution of DTNB (18 mg, 0.045 mmol, in 5 mL of acetonitrile) was added while stirring and the obtained mixture was left overnight. The AuNPs@DTNB were collected by centrifugation (5000 rpm, 10 min) and next purified by six-fold dissolution in acetonitrile and centrifugation procedure and then by ten-fold dissolution in water and centrifugation procedure. The purified AuNPs were dried and suspended in 2.5 mL of water. Further, the 10 µL of solution containing DTNB-modified Au nanoparticles were mixed with 5 µl of 60 µg/ml anti-IL8 solution in PBS buffer and then added to the mixture of the coupling reagents (0.2 M EDC/0.05 M NHS, the volume ratio of 5:1). The resulting mixture was incubated at 4 °C for 4 hours to perform the conjugation reactions. The Raman reporter-labeled immuno-Au-nanoparticles (anti-IL6/AuNPs-FC; anti-IL8/AuNPs-*p*-MBA; anti-IL16/AuNPs-DTNB) were separated from solution by centrifugation at 20000 rpm for 10 min. Then, the suspensions of the Raman reporter-labeled immuno-Au-nanoparticles were passivated with 2,5 µL of 2 % BSA in PBS buffer solution. After 2 h, the mixture was centrifuged again for 10 min at 20000 rpm and then re-suspended in 1 mL PBS solution. Figure S4 shows the sequential steps for the formation of the Raman reporter-labeled immuno-Au nanoparticles. The prepared Raman reporter-labeled immuno-Au-nanoparticles were stored at 4 °C for future use.


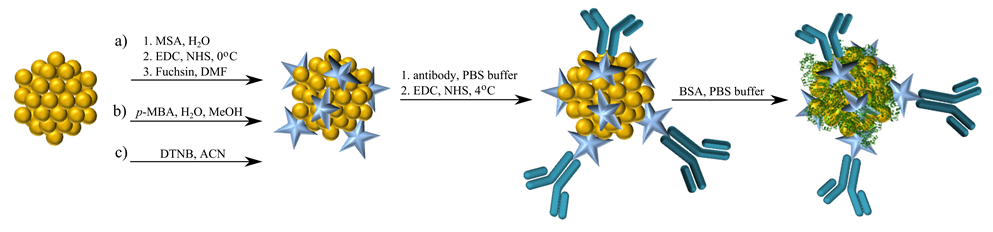


**Fig. S4.** The sequential steps for the formation of the Raman reporter-labeled immuno-Au nanoparticles.

1. **EDC/NHS chemistry.**

The monoclonal antibodies against selected interleukins **(**anti-interleukin 6 (anti-IL6), anti-interleukin-8 (anti-IL8), and anti-interleukin 18 (anti-IL18) were immobilized onto the thiol modified Au SERS-active substrate using the EDC/NHS standard method for coupling amines to carboxylic acids. EDC (1-Ethyl-3-(3-dimethylaminopropyl)-carbodiimide) is a zero-length crosslinking reagent used to couple carboxyl or phosphate groups with primary amines to form amide bonds. N-hydroxysuccinimide (NHS) or N-hydroxysulfosuccinimide (sulfo-NHS) are used to stabilize the intermediate product of this reaction (*O*-acylisourea) by converting it to an amine-reactive NHS ester (Everaerts et al., 2008). NHS ester is considerably more stable than the *O*-acylisourea intermediate, while allowing for efficient conjugation to primary amines at physiological pH. The ability to crosslink primary amines to carboxylic acid groups using EDC/NHS chemistry is a powerful method for crosslinking peptides and proteins, preparing biomolecular probes, and immobilizing macromolecules onto solid substrates.

**Fig. S5.** The UV-visible extinction spectra of AuNPs at different steps: (a) AuNPs as-received, (b) after modification with Raman reporter (DTNB) and (c) after mixing with antibody (anti IL-18 antibodies).

1. **PCA analysis.**

PCA is based on a linear transformation of spectra regarded as N dimensional vectors, where N is the number of data points in a single spectrum, in a new coordinate system in which the new vectors are orthogonal to each other. Each PC represents a linear combination of the original variables (e.g., Raman wavenumber). The first component (in the horizontal direction) is the most important one and accounts for as much variation in the data as possible. In the PCA model, the big spectral set matrix (X) is transformed into two smaller matrices according to the formula X = TP^T^ + E where T is the matrix of scores, P is the matrix of loadings, and E is the error matrix. The PCA method enables one to understand the sources of variation in the obtained SERS data, e.g., the plot of loadings vs. the wavenumber indicates the most important diagnostic vibrations in the spectra.

1. **Microfluidic chip.**

We have performed our experiments using two configurations of the microfluidic chip enable the multiplexed simultaneous (Fig. S6A) and parallel (Fig. S6B) detection of target interleukins.


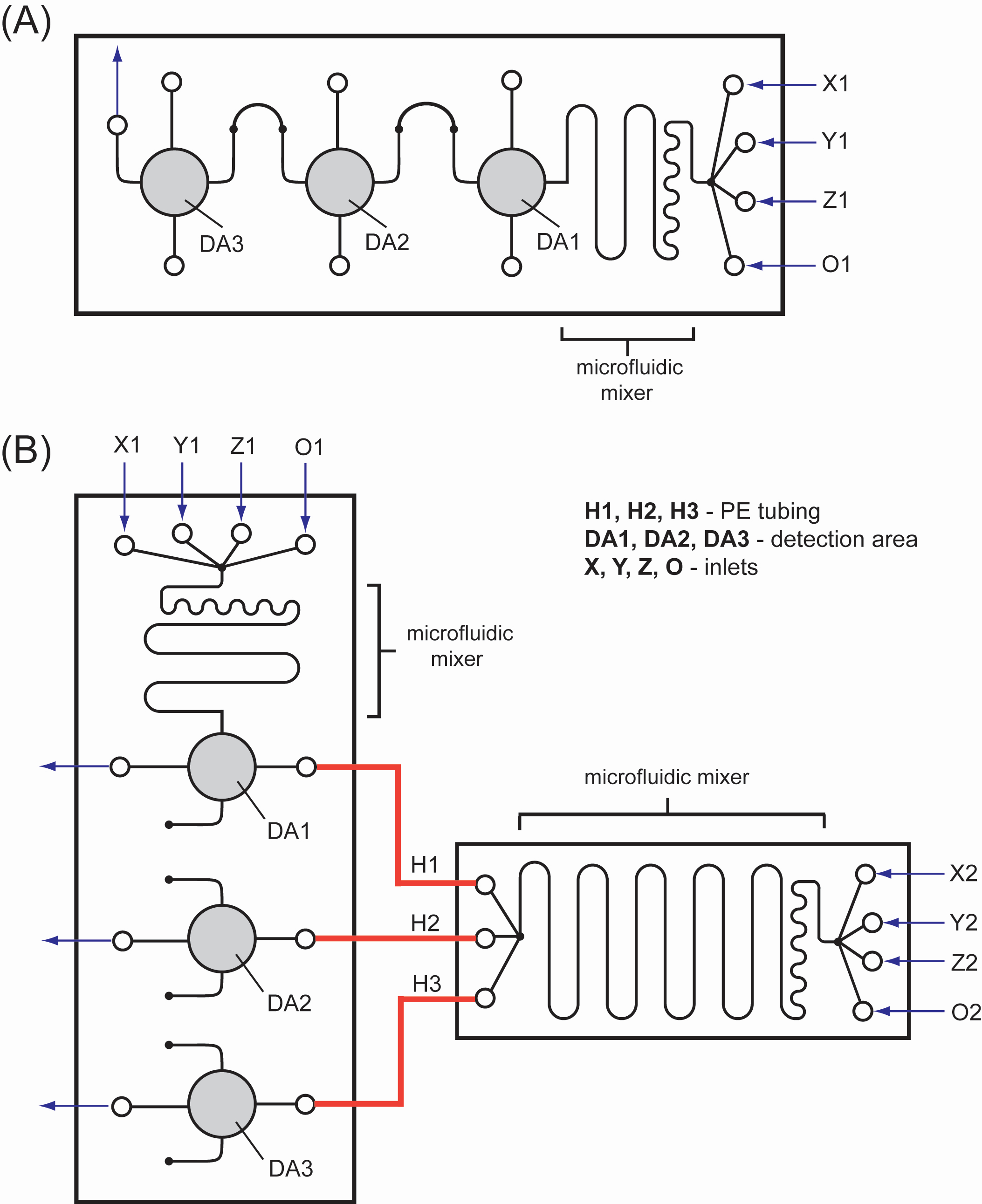


**Fig. S6.** The schematic view of microfluidic chip for: (A) multiplexed simultaneous and (B) parallel detection.

1. **SERS-based IL-6,IL-8, and IL-18 interleukins detection.**


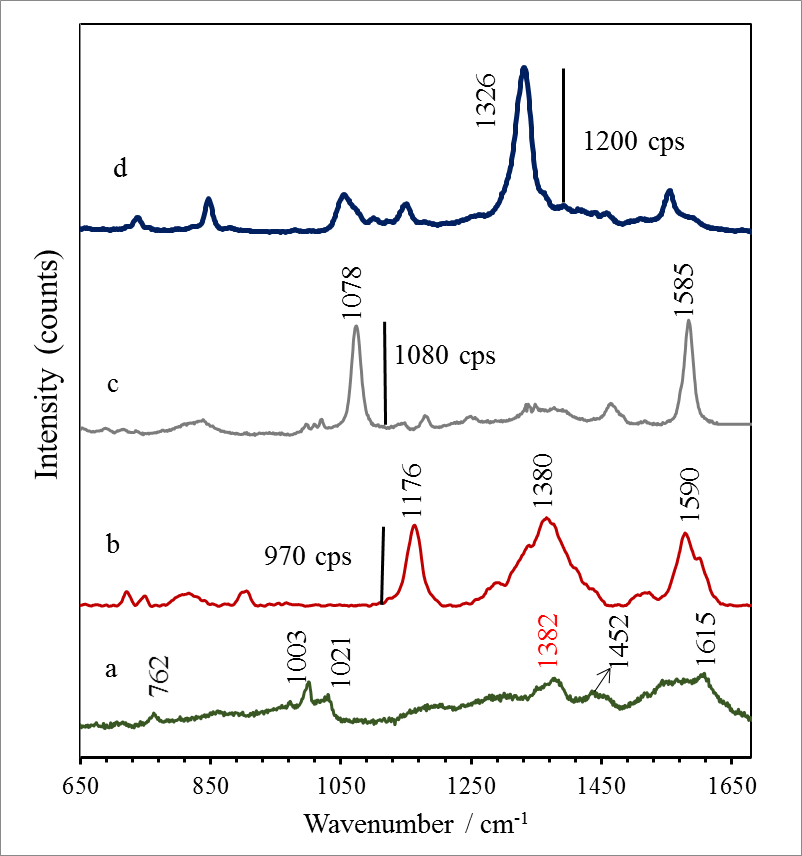


**Fig. S7.** (a) SERS spectrum obtained in the control experiment for 20 ng·ml^-1^ Akt blocking peptide solution in blood plasma; (b, c, and d) SERS spectra in the presence of IL-6, IL-8, and IL-18 in human blood plasma samples at 20 ng·ml^-1^.

**Fig**. **S8.** SERS spectra obtained for increasing concentration of IL-18: (a) 0.0 ng·ml^-1^ in PBS buffer; (b) 0.01; (c) 0.1; 0.1; (e) 0.5; (f) 1.0; (g) 2.5 ng·ml^-1^ in blood plasma.

**Fig. S9.** Reproducibility of three separately prepared SERS immunoassays exposed to different concentrations of IL-18 in blood plasma (0.1 , 0.5, and 30.0 ng/mL). The SERS spectra were recorded from 1 to15 randomly selected spots across the each SERS assay.

| A |
| --- |
| B |

**Fig. S10.** (A) SERS spectra obtained for increasing concentration of IL-8: (a) 0.0; (b) 0.005; (c) 0.01; 0.1; (e) 0.5; (f) 1.0; (g) 2.5; (h) 5.0; (i) 10.0; (j) 30.0 ng/mL in blood plasma. (B) The relationship between the intensity of the marker band at 1326 cm-1 versus the concentration of IL-8 in the range from 0 ng/mL to 30 ng/mL. Each SERS spectrum was averaged from 20 measurements in different places across the SERS surface using mapping mode. The error bars indicate the standard deviations from twenty measurements at different spots for each concentration.


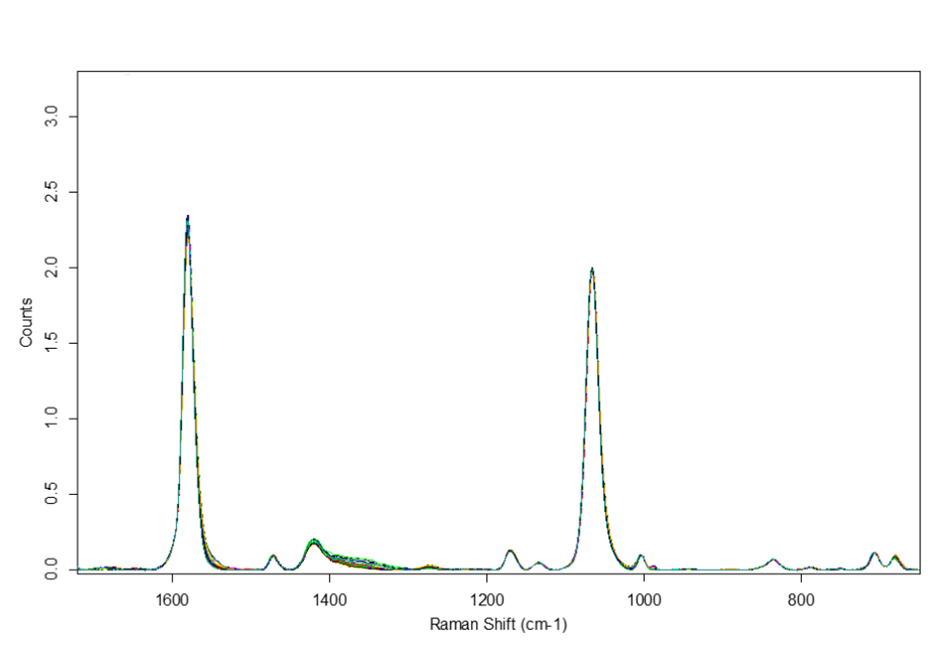


**Fig. S11.** SERS spectra of *p*-MBA adsorbed onto the SERS platform from 2 ng/mL solution of *p*-MBA in human blood. The SERS spectra were recorded from 35 different spots within the same sample. The excitation wavelength was at 632 nm, the laser power was 5 mW, and the acquisition time was 20 s.

| A |
| --- |
| B |

**Fig. S12**. (A) SERS spectra obtained for increasing concentration of IL-6: (a) 0.0; (b) 0.005; (c) 0.01; 0.1; (e) 0.5; (f) 1.0; (g) 2.5; (h) 5.0; (i) 10.0; (j) 30.0 ng/mL in blood plasma. (B) The relationship between the intensity of the marker band at 1326 cm^-1^ versus the concentration of IL-6 in the range from 0 ng/mL to 30 ng/mL. Each SERS spectrum was averaged from 20 measurements in different places across the SERS surface using mapping mode. The error bars indicate the standard deviations from twenty measurements at different spots for each concentration.


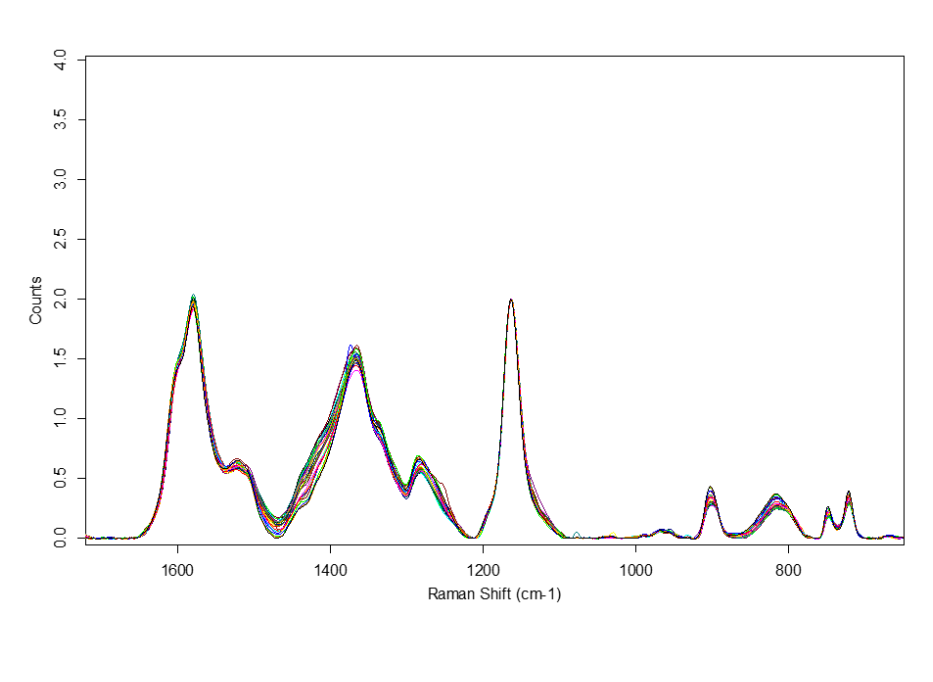


**Fig. S13.** SERS spectra of FC adsorbed onto the SERS platform from 2 ng/mL solution of FC in human blood plasma. The SERS spectra were recorded from 35 different spots within the same sample. The excitation wavelength was at 632 nm, the laser power 5 mW, and the acquisition time was 20 s.


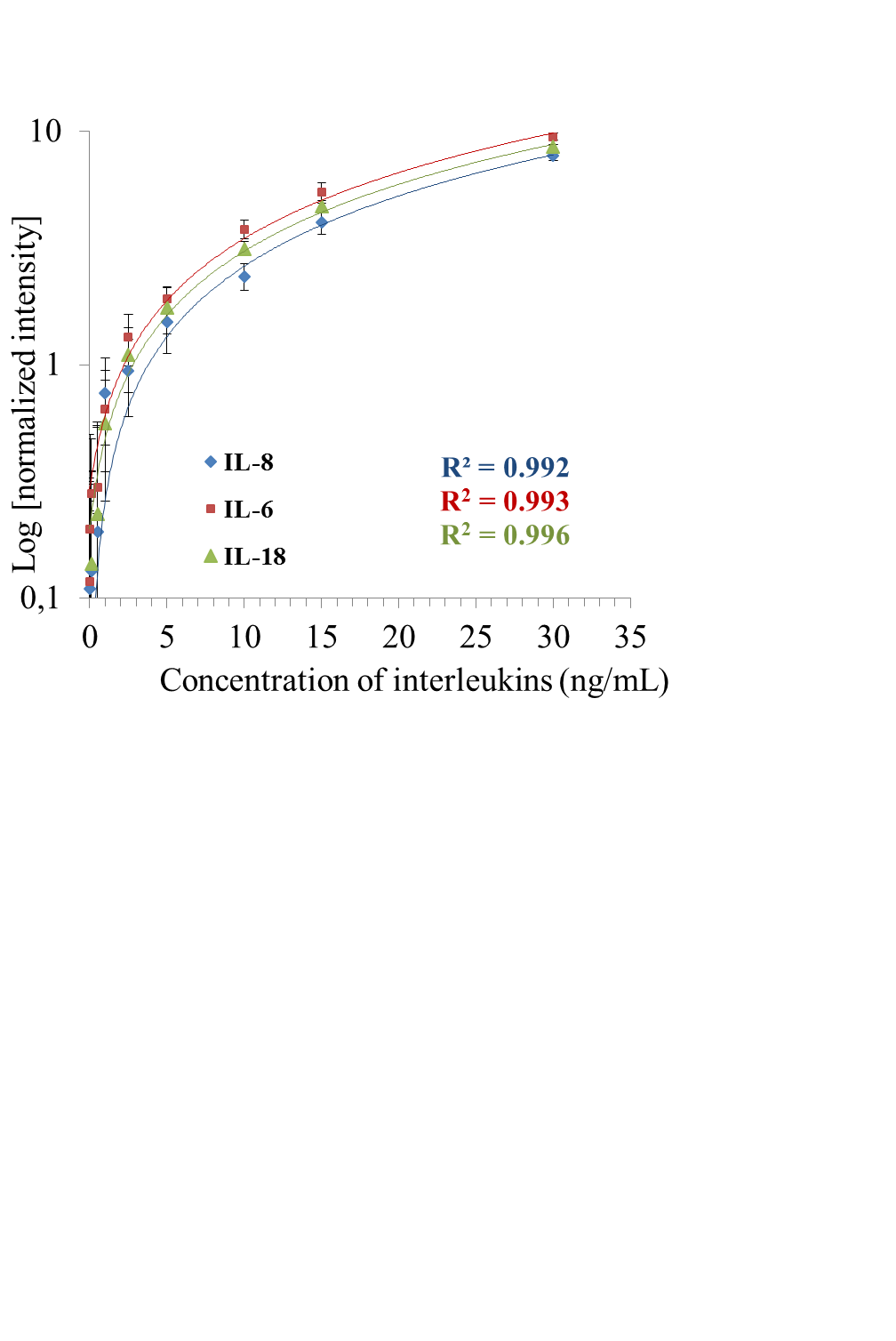


**Fig. S14.** The concentration – intensity calibration curves obtained for simultaneous multiplexed detection of three IL-6, IL-8, and IL-18 interleukins from blood plasma samples. Each SERS spectrum was averaged from 20 measurements at different places across the SERS surface using the mapping mode. The error bars indicate the standard deviations from twenty measurements at different spots for each concentration. Curves were plotted in semi-log plot for better visibility.

| 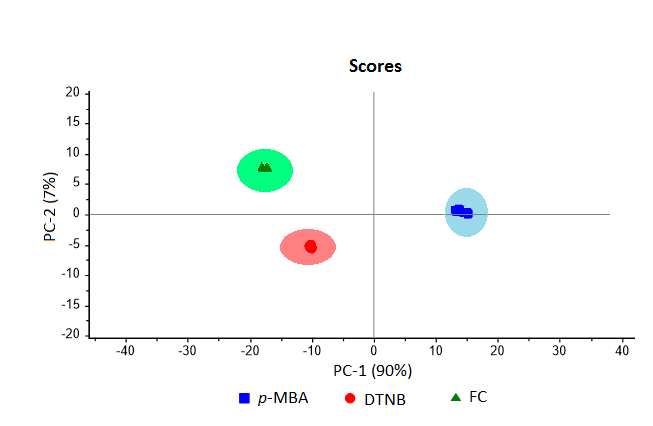A |
| --- |
| 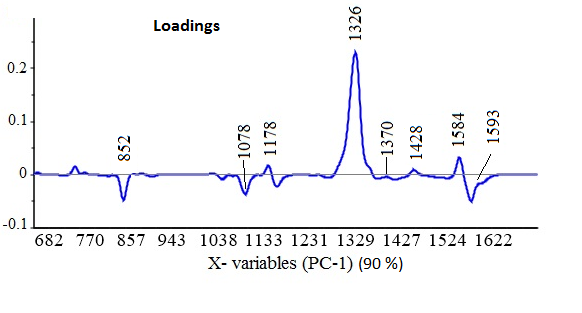B |

**Fig. S15**. (A) PCA scores plots calculated for SERS responses in the multiplexed analysis of three interleukins: IL-6, IL-8, and IL-18 from blood plasma, which were encoded by FC (green cluster), p-MBA (blue clatter), and DTNB (red cluster), respectively. While applying this procedure about 120 spectral responses of these three interleukins of 20 ng·ml-1 concentration in blood plasma samples were processed together. (B) PC-1 loading plot.

REFERENCES

1. Fernandez-Lopez, C.; Mateo-Mateo, C.; Alvarez-Puebla, R. A.; Perez-Juste, J.; Pastoriza-Santos, I.; Liz-Marzan, L. M., Highly Controlled Silica Coating of PEG-Capped Metal Nanoparticles and Preparation of SERS-Encoded Particles. *Langmuir* **2009,** 25, (24), 13894-13899.

2. Enustun, B. V.; Turkevich, J., Coagulation of Colloidal Gold. *Journal of the American Chemical Society* **1963,** 85, (21), 3317-3328.

3. A Sivanesan, E. Witkowska, W. Adamkiewicz, Ł. Dziewit, A. Kamińska and J. Waluk, Analyst, 2014,139, 1037).
